# Supplementary material for: The long noncoding RNA ADIPINT regulates human adipocyte metabolism via pyruvate carboxylase
Source: Nat Commun. 2022 May 26;13:2958. doi: 10.1038/s41467-022-30620-0 (PMC9135762; doi:10.1038/s41467-022-30620-0)
Supplement: Supplementary file 1 — Supplementary Information [file 41467_2022_30620_MOESM1_ESM.pdf]

Supplementary Data Figures

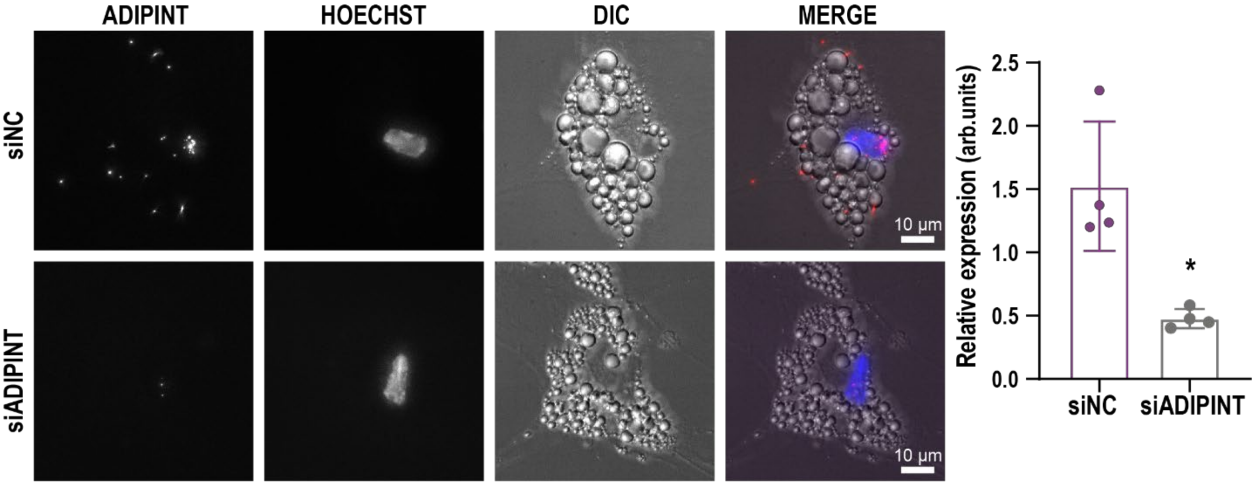

Supplementary Figure 1. ADIPINT RNA FISH signal is depleted upon ADIPINT knockdown
ADIPINT was knocked down using an siRNA and RNA-FISH was performed. FISH signal was compared to control cells (siNC). Hoescht was used to stain for nuclei and differential interference contrast image taken to highlight the lipid droplets and cell boundry (to the right). n = 4 separate images of 10/20 cells per image and quantified (to the left). Unpaired two-sided t-test was used to assess significance. Data are presented as mean values +/- SD. Scale bar is 10 μm. * $p < 0.05$.

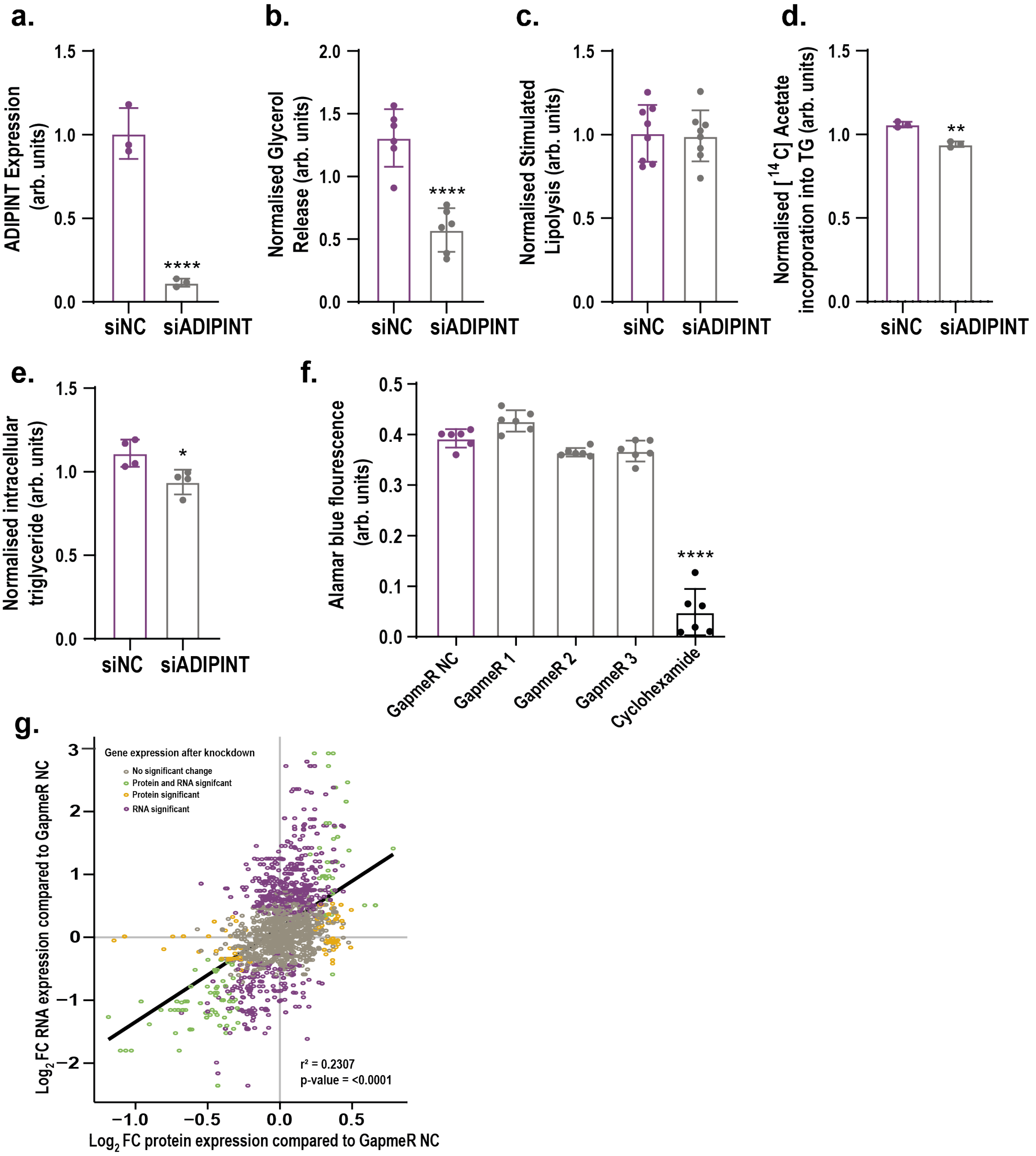

Supplementary Figure 2. Knockdown of ADIPINT decreased lipid metabolism and the aerobic and anaerobic respiration rate in hADSC. a ADIPINT expression (n = 3) **b** basal glycerol release (n = 6) **c** stimulated lipolysis (n = 8) **d** insulin stimulated lipid synthesis (n = 3) and **e** total intracellular triglyceride content (n = 4) after ADIPINT knockdown using a siRNA. A two-sided unpaired t-test was used to assess significance. **f** Cell viability as measured by Alamar blue fluorescence after ADIPINT knockdown using GapmeRs 1-3 (n = 6). Each data point represents an independent experiment, one way ANOVA with Dunnett's post-test was used to assess significance. Data are presented as mean values +/- SD. **g** A scatter plot of log2 fold change (FC) in RNA plotted against the log2 fold change in protein for each gene detected at both the RNA and protein level, after ADIPINT knockdown. The median fold change across the three ADIPINT-targeting GapmeRs (1-3) compared to GapmeR NC is plotted and the median false discovery rate (FDR) < 0.05 was used to determine significance. All genes are plotted except Transferrin due to the high RNA log2FC (6.6). No change was observed at the protein level. Linear regression was used to assess significance between global changes in RNA and protein. Error bars are SD. * $p < 0.05$, ** $p < 0.01$, **** $p < 0.0001$.

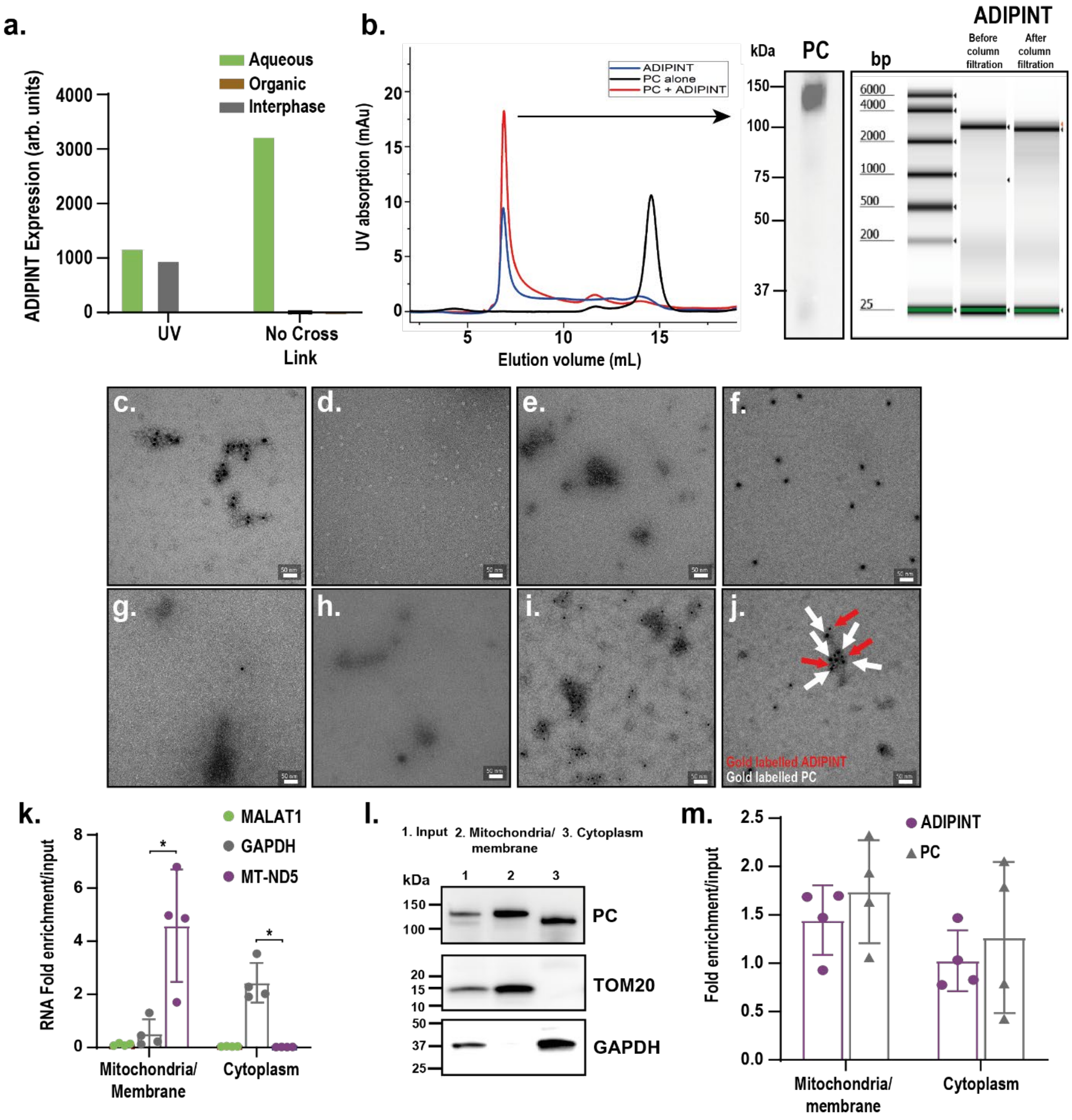

Supplementary Figure 3. ADIPINT and PC interact and are located in the same cellular fractions a hADSC cells underwent UV crosslinking before collection in Trizol. After Trizol-chloroform seperation the aqueous and organic phase was collected. The interphase was subjected to two further rounds of Trizol-chloroform speration before collection. ADIPINT expression was analyzed in each fraction and a shift into the interphase was seen after UV crosslinking. **b.** Purified PC (black), purified ADIPINT (blue) and PC with ADIPINT were injected onto a sepharose column and UV absorbtion measured. PC alone gave a peak at 14.56 mL (termed PC elution) and ADIPINT a peak at 6.87 mL (termed RNA elution). The peak at 6.87 mL for the PC with ADIPINT injection was collected for protein (to the left) and RNA analysis. Western blot analysis revealed PC protein was present in the RNA elution peak alongside ADIPINT (to the right). ADIPINT before injection onto the column was run alongside ADIPINT recovered after column filtration demonstarting ADIPINT remained intact. Negative staining of **c** ADIPINT and PC labelled with 20 nm gold beads **d** PC alone (before gel filtration) **e** ADIPINT alone (before gel filtration) **f** PC alone labelled with 20nm gold beads **g** ADIPINT alone labelled with 20nm gold beads **h** ADIPINT anti-sense and PC labelled with 20nm gold beads **i** ADIPINT labelled with 10nm gold beads **j** ADIPINT labelled with 10 nm gold beads and PC labelled with 20 nm gold beads. White arrows correspond to 20 nm gold beads and red arrows to 10 nm gold beads. Scale bar is 50 nm. **k** qRT-PCR analysis of MALAT1 (nuclear), GAPDH (cytoplasmic) and MT-

ND5 (Mitochondrial encoded) RNA after ultracentrifugation to enrich the mitochondrial/membrane and cytoplasmic fractions (n = 4). One way ANOVA with Tukey's post-test was used to assess significance. Significance between GAPDH and MT-ND5 is shown. **l** Western blot analysis of PC,TOM20 and GAPDH after ultracentrifugation to enrich the mitochondrial/membrane and cytoplasmic fractions. **m** Quantification of PC and ADIPINT in the mitochondria/membrane and cytoplasmic fractions compared to the input sample using western blot and qRT-PCR respectively (n = 4). **k,m** each data point represents an individual experiment. Data are presented as mean values +/- SD. * $p < 0.05$

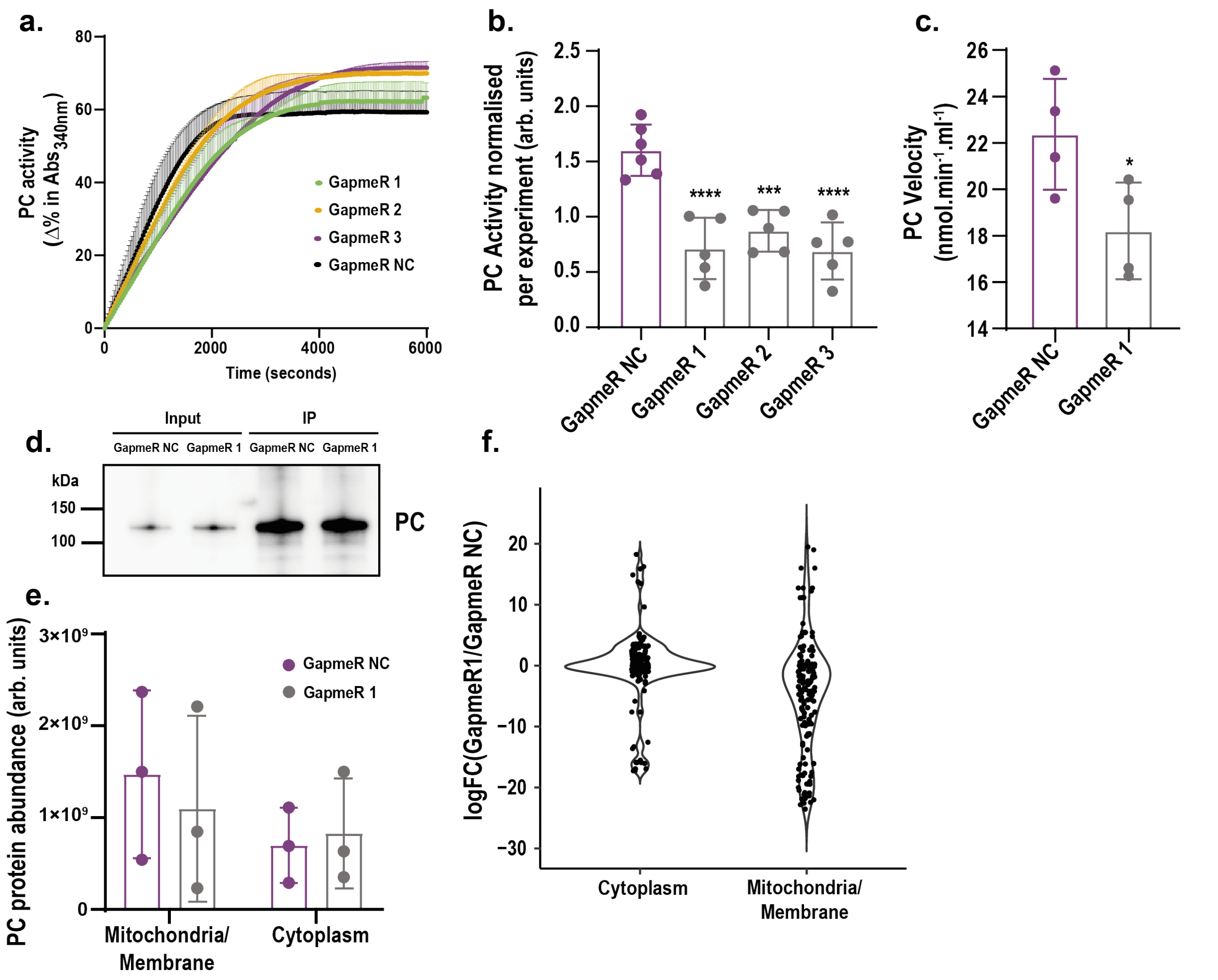

Supplementary Figure 4. Pyruvate carboxylase activity is decreased after ADIPINT knockdown
a PC activity measured through a change in absorbance of NADH (340 nm) after transfection with GapmeR NC (black) or ADIPINT-targeting GapmeRs 1-3. The lines represent three independent experiments assayed at the same time. No differences in the total amount of substrate metabolized by PC was detected across all experiments. n = 3 independent experiments **b** PC activity assesed by the time to reach 50% completion for the reaction (seconds) was normalised to the average time across all conditions for each experiment and plotted (n = 5-6). GapmeR 1, 2 and 3 targeting ADIPINT significantly decreased PC activity. **c** PC initial velocity measured in the mitochondrial fraction after ADIPINT knockdown using GapmeR 1 (n = 4). **d** Western blot for PC in GapmeR 1 and GapmeR NC transfected cells after PC immunoprecipitation (IP). IP samples were compared to input samples from the same experiment. **e** PC protein abundance measured using LC/MS of PC immunoprecipitation samples of GapmeR 1 and GapmeR NC transfected cells. No significantdifferences were detected in the amount of PC in the two conditions in the mitochondria/membrane or cytoplasmic fraction following IP (n = 3). **f** The distribution of the log2 fold changes for proteins after PC immunoprecipitation in the mitochondrial and cytoplasmic fractions following ADIPINT knockdown with GapmeR 1. Two-tailed t-test was used to compare significance between GapmeR 1 and Gapmer NC samples. One-way ANOVA with Dunnett post-test comparing each GapmeR to GapmeR NC was used to assess significance. **b-c**, **e** each data point represents an individual experiment, mean is plotted and error bars are SD. * $p < 0.05$, *** $p < 0.001$, **** $p < 0.0001$.

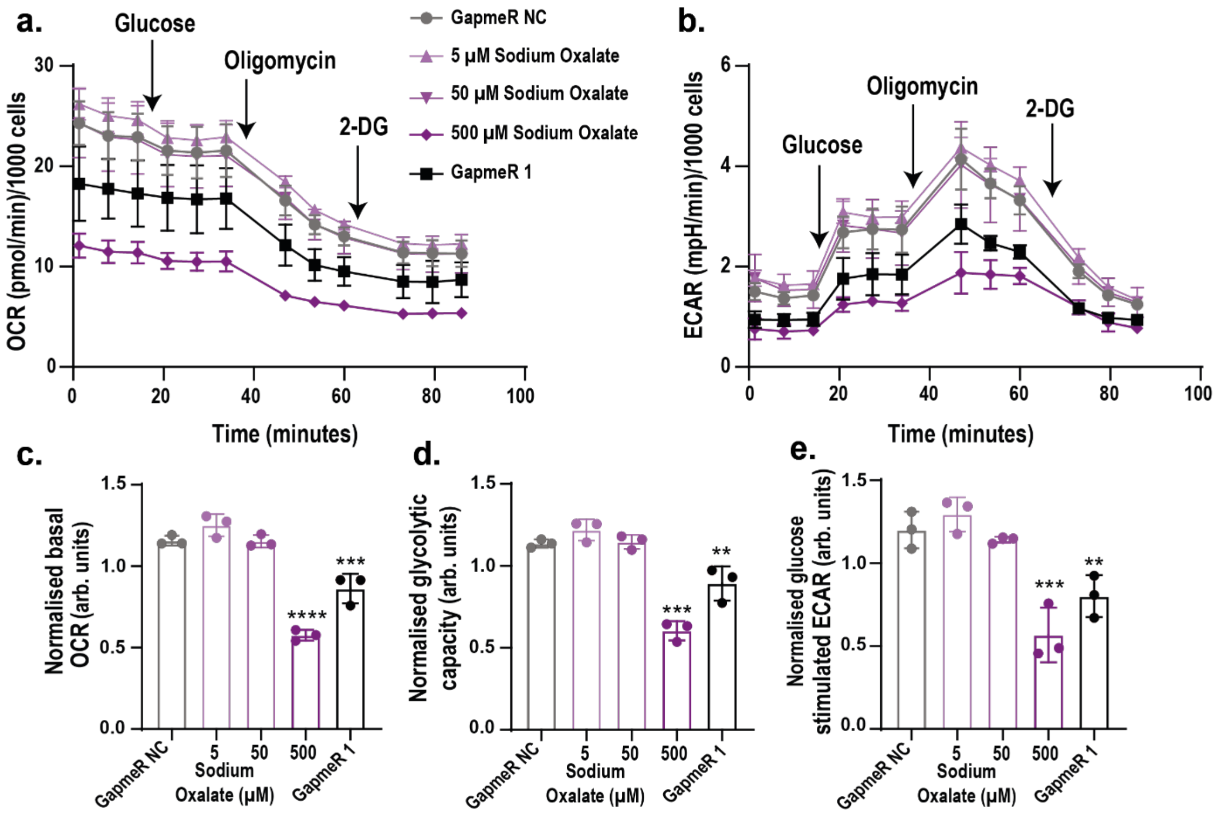

Supplementary Figure 5. Inhibition of pyruvate carboxylase decreases the aerobic and anaerobic respiration rate in hADSC. Sodium Oxalate (5-500 µM) was used to inhibit pyruvate carboxylase and the **a** oxygen consumption rate (OCR) and **b** extracellular acidification rate (ECAR) was measured at basal and then after glucose, oligomycin and 2-deoxyglucose treatment as indicated. GapmeR NC cells were used as a control and GapmeR 1 treated cells used as a positive control for reductions in OCR and ECAR n. The **c** basal OCR, **d** glycolytic capacity and **e** glucose-stimulated ECAR in hADSC after oxalate administration. n = 3 independent experiments per group. One-way ANOVA with Dunnett's post-hoc test assessed significance between GapmeR NC versus GapmeR NC plus oxalate treatment and GapmeR 1. Data are presented as mean values +/- SD. ** $p < 0.01$, *** $p < 0.001$, **** $p < 0.0001$.

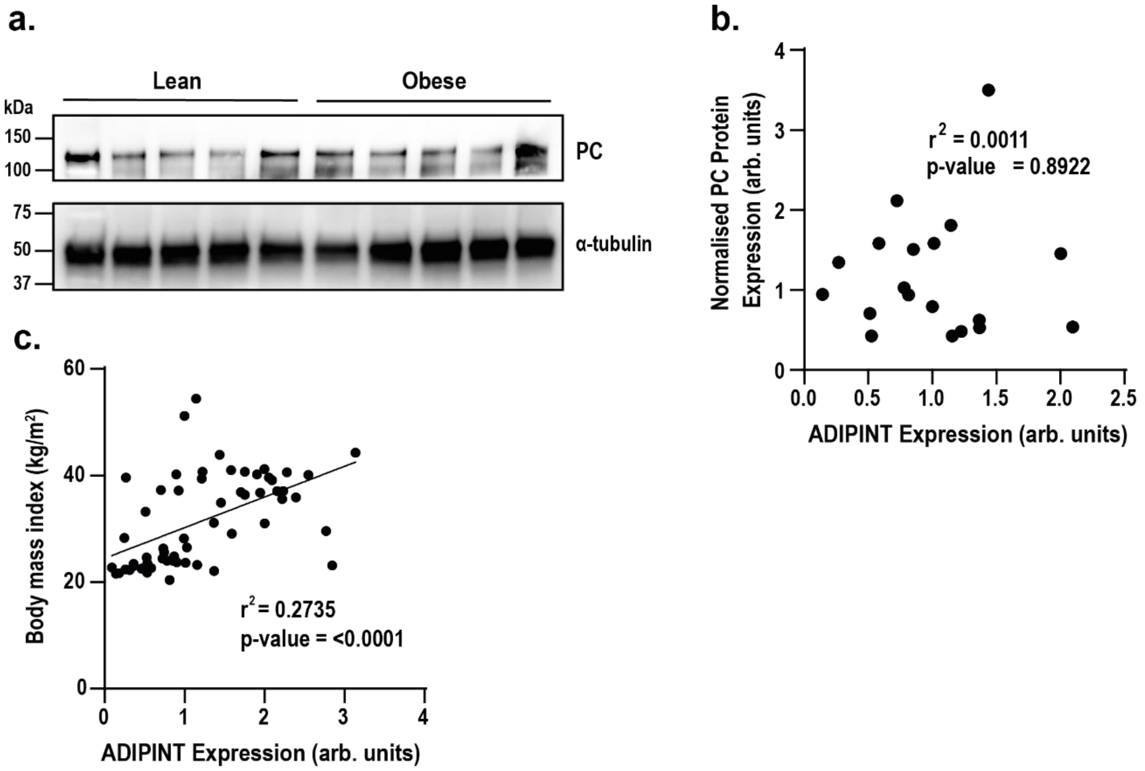

Supplementary Figure 6. PC protein expression in WAT from lean and obese patients plotted with ADIPINT expression a Representative western blot of PC and α-tubulin protein expression in WAT, showing 5 lean and 5 obese patient samples. The same volume of WAT lysate loaded into the PC activity assay was used for western blot analysis. **b** PC protein expression from the lean and obese patients is plotted against ADIPINT expression measured in the same sample. No significant correlation was observed. **c** ADIPINT expression plotted against body mass index from patients in cohort 2 and 3. Each data point represents one individual patient. Linear regression was used to assess significance.

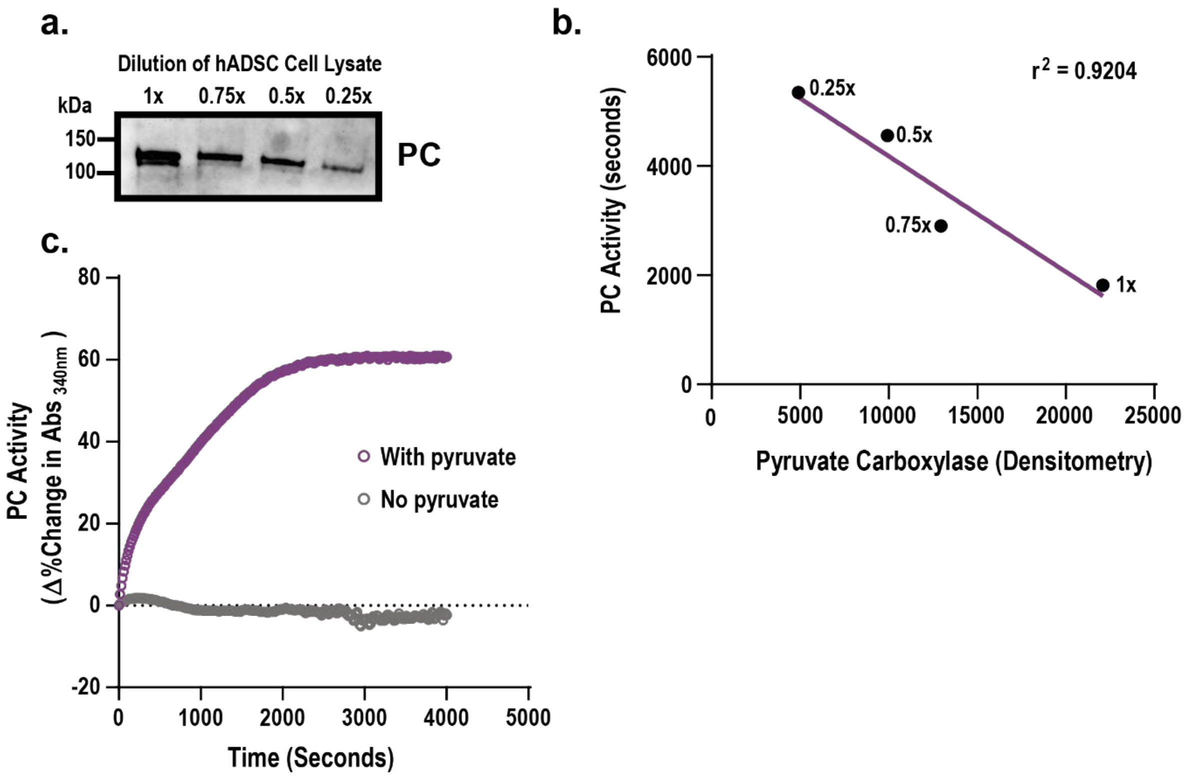

Supplementary Figure 7 Pyruvate carboxylase protein expression and enzymatic activity share a linear relationship a Western blot analysis of PC after serial dilution of hADSC cell lysate from 1x to 0.25x. **b** PC activity (representing the time taken for 50% of the PC activity assay to be completed) for each dilution of hADSC cell lysate plotted against the PC protein expression as shown in **a**. **c** PC activity assay with hADSC lysate added with and without pyruvate.

Supplementary Methods Tables

Supplementary Method Table 1. List of Antisense LNA GapmeRs/siRNA used for knockdown		
Target Gene	Identifier	Sequence
ADIPINT	GapmeR 1	TCTTGATTGCTGCAGA
ADIPINT	GapmeR 2	TACTTTGCCTCTTAGA
ADIPINT	GapmeR 3	CGAAGATTCATGGTCA
Negative Control A	GapmeR NC	Purchased from Qiagen as Negative Control A Cat# LG00000002
ADIPINT	siADIPINT	GAACAGGACUGCAAAGAAA
siGENOME Non-targeting siRNA #3	siNC	AUGUAUUGGCCUGUAUUAG
Pyruvate carboxylase	siPC	GAGCUGAUGUGGUGGAUGU

Supplementary Method Table 2. List of primer sequences for target genes			
Target Gene	Direction	Sequence	
ADIPINT	Forward	TTTCCGCAGGGATTACTTTG	
ADIPINT	Reverse	TCCTTGACTACGGGCAATTC	
Pyruvate Carboxylase	Forward	AGTTCAAGGAGGTCAAGAA	
Pyruvate Carboxylase	Reverse	ATCCATTCTGCACCATAAAC	
β2-Microglobulin	Forward	AAGGACTGGTCTTTCTATCTC	
β2-Microglobulin	Reverse	GATCCCACTTAACTATCTTGG	
GAPDH	Forward	ACAGTTGCCATGTAGACC	
GAPDH	Reverse	TTTTTGGTTGAGCACAGG	
18S	Forward	TGACTCAACACGGGAAACC	
18S	Reverse	TCGCTCCACCAACTAAGAAC	
MT-ND5	Forward	ACATCTGTACCCACGCCTTC	
MT-ND5	Reverse	TCGATGATGTGGTCTTTGGA	
NEAT1	Forward	GCTACAAGGTGGGGAAGACT	
NEAT1	Reverse	AGTCTGACGCCCATCTTTCA	
U1	Forward	GGGAGATACCATGATCACGAAGGT	
U1	Reverse	CCACAAATTATGCAGTCGAGTTTCCC	

Supplementary Method Table 3. List of biotinylated oligo sequences used for TROOPS

Gene Target	Sequence	Designation			
ADIPINT	aagtaatccctgcggaaact	Odd			
ADIPINT	ttgactacgggcaattcacg	Even			
ADIPINT	agacagggtcagagtctgag	Odd			
ADIPINT	gtgcatccgggaactaaatc	Even			
ADIPINT	aaggtagaccacaggaggag	Odd			
ADIPINT	ctctgacattcaaggtctca	Even			
ADIPINT	tcagtgggaagctatcactg	Odd			
ADIPINT	actgagctttcagaggacta	Even			
ADIPINT	caacccttttattattcctc	Odd			
ADIPINT	gaaacaagttctcgtcctgg	Even			
ADIPINT	gacagggggatgtcagagac	Odd			
ADIPINT	ctaaacggctctgtggtctg	Even			
ADIPINT	gcaattcctcattccaaaca	Odd			
ADIPINT	ctgcaatcttggttgacaca	Even			
ADIPINT	aaacttgtatatgacggccc	Odd			
ADIPINT	tggctgttaaagagcttacc	Even			
ADIPINT	tatgtggtcttgggcacaaa	Odd			
ADIPINT	tattattcctcctacgtgta	Even			
ADIPINT	cattggaagggcaggagagc	Odd			
ADIPINT	tctccattagcgtaacatgt	Even			
ADIPINT	agtagccacagccacaaaga	Odd			
ADIPINT	tgactgatgggagctgggaa	Even			
ADIPINT	atattcttggtctgtgcttt	Odd			
ADIPINT	ctctcttatatctgtgacca	Even			
Lac Z	ccagtgaatccgtaatcatg	n/a			
Lac Z	tcacgacgttgtaaaacgac	n/a			
Lac Z	attaagttgggtaacgccag	n/a			
Lac Z	aggttacgttggtgtagatg	n/a			
Lac Z	aatgtgagcgagtaacaacc	n/a			
Lac Z	gtagccagctttcatcaaca	n/a			
Lac Z	aataattcgcgtctggcctt	n/a			
Lac Z	agatgaaacgccgagttaac	n/a			
Lac Z	aattcagacggcaaacgact	n/a			
Lac Z	tttctccggcgcgtaaaaat	n/a			
Lac Z	atcttccagataactgccgt	n/a			
Lac Z	aacgagacgtcacggaaaat	n/a			
Lac Z	gctgatttgtgtagtcggtt	n/a			
Lac Z	ttaaagcgagtggcaacatg	n/a			
Lac Z	aactgttacccgtaggtagt	n/a			
Lac Z	ataatttcaccgccgaaagg	n/a			
Lac Z	tttcgacgttcagacgtagt	n/a			
Lac Z	atagagattcgggatttcgg	n/a			
Lac Z	accattttcaatccgcacct	n/a			
Lac Z	ttaacgcctcgaatcagcaa	n/a			
Lac Z	ttcatcagcaggatatcctg	n/a			
Lac Z	cacggcgttaaagttgttct	n/a			
Lac Z	tggttcggataatgcgaaca	n/a			
Lac Z	ttggcttcatccaccacata	n/a			
