# Supplementary material for: The long noncoding RNA ADIPINT regulates human adipocyte metabolism via pyruvate carboxylase
Source: Nat Commun. 2022 May 26;13:2958. doi: 10.1038/s41467-022-30620-0 (PMC9135762; doi:10.1038/s41467-022-30620-0)
Supplement: Supplementary file 3 — Description of Additional Supplementary Information [file 41467_2022_30620_MOESM3_ESM.pdf]

The long noncoding RNA ADIPINT regulates human adipocyte metabolism via pyruvate carboxylase

Supplementary Files provided

Supplementary Figures 1-7

Supplementary Methods Tables 1-3

Supplementary Data 1-15
