# Supplementary material for: The long noncoding RNA ADIPINT regulates human adipocyte metabolism via pyruvate carboxylase
Source: Nat Commun. 2022 May 26;13:2958. doi: 10.1038/s41467-022-30620-0 (PMC9135762; doi:10.1038/s41467-022-30620-0)
Supplement: Supplementary file 19 — Reporting Summary [file 41467_2022_30620_MOESM19_ESM.pdf]

nature research

Corresponding author(s): Arner P, Gao H

Last updated by author(s): Aug 20, 2020

Reporting Summary

Nature Research wishes to improve the reproducibility of the work that we publish. This form provides structure for consistency and transparency in reporting. For further information on Nature Research policies, see our Editorial Policies and the Editorial Policy Checklist.

Statistics

For all statistical analyses, confirm that the following items are present in the figure legend, table legend, main text, or Methods section.

n/a	Confirmed	
☐	☒	The exact sample size (*n*) for each experimental group/condition, given as a discrete number and unit of measurement
☐	☒	A statement on whether measurements were taken from distinct samples or whether the same sample was measured repeatedly
☐	☒	The statistical test(s) used AND whether they are one- or two-sided<br>*Only common tests should be described solely by name; describe more complex techniques in the Methods section.*
☒	☐	A description of all covariates tested
☐	☒	A description of any assumptions or corrections, such as tests of normality and adjustment for multiple comparisons
☐	☒	A full description of the statistical parameters including central tendency (e.g. means) or other basic estimates (e.g. regression coefficient) AND variation (e.g. standard deviation) or associated estimates of uncertainty (e.g. confidence intervals)
☐	☒	For null hypothesis testing, the test statistic (e.g. *F*, *t*, *r*) with confidence intervals, effect sizes, degrees of freedom and *P* value noted<br>*Give P values as exact values whenever suitable.*
☒	☐	For Bayesian analysis, information on the choice of priors and Markov chain Monte Carlo settings
☒	☐	For hierarchical and complex designs, identification of the appropriate level for tests and full reporting of outcomes
☐	☒	Estimates of effect sizes (e.g. Cohen's *d*, Pearson's *r*), indicating how they were calculated

Our web collection on statistics for biologists contains articles on many of the points above.

Software and code

Policy information about availability of computer code

Data collection	No software was used
Data analysis	Qlucore Omics Explorer v 3.4<br>Bioconductor R package Limma v 3.40.6<br>GraphPad Prism v 8.0<br>ImageJ (Fiji) v 1.51<br>Mascot v 2.5.1

For manuscripts utilizing custom algorithms or software that are central to the research but not yet described in published literature, software must be made available to editors and reviewers. We strongly encourage code deposition in a community repository (e.g. GitHub). See the Nature Research guidelines for submitting code & software for further information.

Data

Policy information about availability of data

All manuscripts must include a data availability statement. This statement should provide the following information, where applicable:

- Accession codes, unique identifiers, or web links for publicly available datasets
- A list of figures that have associated raw data
- A description of any restrictions on data availability

Gene expression microarray data have been deposited at the NCBI Gene Expression Omnibus (GEO) and are identified by accession numbers GSE199076 and GSE199063. Proteomics data are available via ProteomeXchange with identifier PXD032769. Figures 1-6 and Supplementary Figures 1-6 all raw data is provided in the Source Data File. Other data are available from the corresponding authors upon request.

Field-specific reporting

Please select the one below that is the best fit for your research. If you are not sure, read the appropriate sections before making your selection.

☒ Life sciences ☐ Behavioural & social sciences ☐ Ecological, evolutionary & environmental sciences

For a reference copy of the document with all sections, see nature.com/documents/nr-reporting-summary-flat.pdf

Life sciences study design

All studies must disclose on these points even when the disclosure is negative.

Sample size	For clinical cohort the sample size was calculated from data on fat cell size in the cohort published PMID : 27852664<br>For all cell-based and molecular studies n=4-6 replicates are commonly used for an α=0.05 and β=0.2 to detect a 30% difference. Based on this n=4-6 was chosen for this study. For protein identification studies such as TROOPS an n=3 was used as the % difference is assumed to be larger than 30%.
Data exclusions	No data was excluded
Replication	The identification of clinically relevant lncRNA took place by examining two separate clinical cohorts collated and examined at different times Experiments carried out in the human adipose derived stem cell line were carried out in different frozen stocks across different passages of the cells. All experiments presented could be replicated
Randomization	Not relevant as clinical study was observational
Blinding	Work in human adipose derived stem cells was not blinded as experiments were performed and analyzed by the same person. In addition there was no subjective analyses (data was generated by analytical machines).

Reporting for specific materials, systems and methods

We require information from authors about some types of materials, experimental systems and methods used in many studies. Here, indicate whether each material, system or method listed is relevant to your study. If you are not sure if a list item applies to your research, read the appropriate section before selecting a response.

Materials & experimental systems

n/a	Involved in the study	
☐	☒	Antibodies
☐	☒	Eukaryotic cell lines
☒	☐	Palaeontology and archaeology
☒	☐	Animals and other organisms
☐	☒	Human research participants
☐	☒	Clinical data
☒	☐	Dual use research of concern

Methods

n/a	Involved in the study	
☒	☐	ChIP-seq
☒	☐	Flow cytometry
☒	☐	MRI-based neuroimaging

Antibodies

Antibodies used	PC (PA5-50101, ThermoFisher)<br>PC (16588-1-AP, ProteinTech)<br>IgG (PP64B, Millipore)<br>α-tubulin (2144, CST)<br>GLS (ab200408, Abcam)<br>GAPDH (14C10, CST)<br>TOM20 (D8T4N, CST)<br>α-rabbit IgG, HRP-linked antibody (7074, CST)<br>α-mouse IgG, HRP-linked Antibody (7076, CST)<br>Digoxin Monoclonal Antibody (25P1C9, ThermoFisher)
Validation	All antibodies were validated by the suppliers and accurately represent expected expression patterns.<br>PC (PA5-50101) - Validated for WB, IHC IF and ICC by supplier. Knockdown of PC using siRNA was validated in house.<br>PC (16588-1-AP) - Validated for WB, IP IHC and IF by supplier including using siRNA against PC and WB. Knockdown of PC using siRNA was validated and presented in the manuscript Fig. 5h. Antibody was used for IP and was validated using mass-spectrometry.<br>IgG - Polyclonal rabbit with over 100 citations including for use as a control in IP experiments (PMID: 24076601).<br>α-tubulin - 365 citations. Validated for WB, IHC, IF and FC by supplier<br>GLS - 2 citations. Validated for WB by supplier using GLS KO cell line.<br>GAPDH - 3261 citations. Validated for WB, IP and IHC by supplier. Displayed expected expression pattern in cell fractionation studies

carried out.
TOM20 - 30 citations. Validated for WB, IP, IHC and IF by supplier Displayed expected expression pattern in cell fractionation studies carried out.
Digoxin - Validated for WB, IP and ELISA by supplier. Validated by us to bind digoxin labelled ADIPINT using electron microscopy.

Eukaryotic cell lines

Policy information about cell lines

Cell line source(s)	Cell line was derived in house from the stromal vascular fraction of subcutaneous adipose tissue from a male donor. The cell line has been described in detail in the following publications PMID: 28470788 PMID: 23393180
Authentication	The cell line was developed, cultured and passaged all in house by dedicated cell culture technicians and authors of the manuscript. The cells are regularly assessed for phenotype and transcriptomic profiles.
Mycoplasma contamination	Cell line tested negative for mycoplasma
Commonly misidentified lines (See ICLAC register)	None

Human research participants

Policy information about studies involving human research participants

Population characteristics	This is third study on Cohort 1 (PMID: 27852664), detailed clinical data is listed in Supplementary Table 1 and Cohort 2 and 3 clinical data is listed in Supplementary Table 14 .
Recruitment	Patients were admitted for bariatric surgery and all asked for participation in the experimental studies involving fat biopsies, all who agreed were included.
Ethics oversight	Approved by Local Ethics Committee in Stockholm (Regional etikprövningsnämnden i Stockholm, Karolinska Institutet Tomtebodavägen 18A 17165 Solna, Sweden)

Note that full information on the approval of the study protocol must also be provided in the manuscript.

Clinical data

Policy information about clinical studies
All manuscripts should comply with the ICMJE guidelines for publication of clinical research and a completed CONSORT checklist must be included with all submissions.

Clinical trial registration	NCT01785134
Study protocol	Available at NCT01785134 and regional ethics board
Data collection	The first patient was included 2006 and the last one included 2010 they were re-examined two and five years post bariatric surgery.
Outcomes	The primary outcome was changes in fat mass and fat cell size, the secondary outcomes was changes in long non-coding RNA in white adipose tissue biopsies
